# An Overview of Virulence, Resistance, and Clinical Significance of Klebsiella Infections

**DOI:** 10.7759/cureus.108688

**Published:** 2026-05-11

**Authors:** Pratignya G Biradar, Harsha V Patil, Satish R Patil

**Affiliations:** 1 Department of Microbiology, Krishna Institute of Medical Sciences, Krishna Vishwa Vidyapeeth (Deemed to be University), Karad, IND

**Keywords:** antimicrobial resistance, carbapenem resistance, esbl, klebsiella pneumoniae, multidrug resistance, nosocomial infections, virulence factors

## Abstract

Gram-negative, encapsulated, non-motile bacilli called *Klebsiella* species are commonly found in the environment and are a natural component of both human and animal flora. Among the genus, *Klebsiella pneumoniae* and *Klebsiella oxytoca* are the most clinically significant, while emerging species such as *Klebsiella variicola* and *Klebsiella aerogenes* are increasingly recognized in human infections. These organisms act as opportunistic pathogens and are associated with a variety of community-acquired and nosocomial infections, particularly in immunocompromised and hospitalized patients. Common clinical manifestations include urinary tract infections, pneumonia, bloodstream infections, wound and soft-tissue infections, intra-abdominal infections, and meningitis.

The pathogenicity of *Klebsiella* species is attributed to multiple virulence factors, including a prominent polysaccharide capsule, adhesins, siderophores, lipopolysaccharide, and the ability to form biofilms. In recent years, the clinical importance of these organisms has grown as a result of the global emergence of multidrug-resistant (MDR) strains. The production of extended-spectrum β-lactamases (ESBLs), AmpC β-lactamases, and carbapenemases has significantly reduced the effectiveness of commonly used antibiotics. Furthermore, MDR-hypervirulent (MDRhv) clones, which are linked to serious infections and few treatment choices, have emerged as a result of the convergence of multidrug resistance and hypervirulence. Accurate laboratory diagnosis and testing for antibiotic susceptibility are crucial for appropriate management and control of these infections.

## Introduction and background

*Klebsiella *spp. are ubiquitous in nature and can be found in environmental samples (surface water, sewage, soil, and plants) and also colonize the mucosal surfaces of healthy mammals [1]. The major species of this genus is *Klebsiella pneumoniae*, followed by *Klebsiella oxytoca*, both considered as opportunistic pathogens with major relevance in community- and hospital-acquired (nosocomial) infections, which are particularly severe in immunocompromised subjects such as those hospitalized in transplant, intensive care (ICU), or neonatal units (NICU) [1].

Digestive tract infections, nosocomial infection outbreaks, hospital-acquired surgical wound infections, and community-onset infections are caused by this prevalent pathogen [2]. It is widely distributed in the environment and acts as an opportunistic pathogen. Klebsiella species can cause a variety of both nosocomial and community-acquired infections, including respiratory tract infections, wound and soft tissue infections, and urinary tract infections (UTIs) [3]. Several clinically significant species, including *Klebsiella pneumoniae, Klebsiella ozaenae, Klebsiella rhinoscleromatis, Klebsiella oxytoca, *and* Klebsiella aerogenes*, are members of this genus. The two major pathotypes, hypervirulent (hv) and multidrug-resistant (MDR) strains, contribute significantly to the burden of *Klebsiella* infections [2].

Because the two groups of strains have distinct genetic backgrounds, they were regarded as non-overlapping. However, it has been demonstrated that *Klebsiella *species can acquire genetic components and mutations that give antibiotic resistance and/or virulence features. This eventually results in the creation of convergent clones known as MDR and multidrug-resistant hypervirulent (MDRhv) *Klebsiella *spp. MDRhv strains exhibit both hypervirulence and multidrug resistance characteristics in *Klebsiella *species and continue to evolve into strains with diverse phenotypes. Numerous MDR-hv *Klebsiella *species strains that have developed via various methods have been recorded in numerous publications from different continents worldwide. Because of the increase in serious infections and the rising absence of effective therapies, *Klebsiella *bacteria that are MDR-hv have evolved into true superbugs that present grave dangers to the general public's health [2].

Methods

Research Strategy 

**Figure 1:**
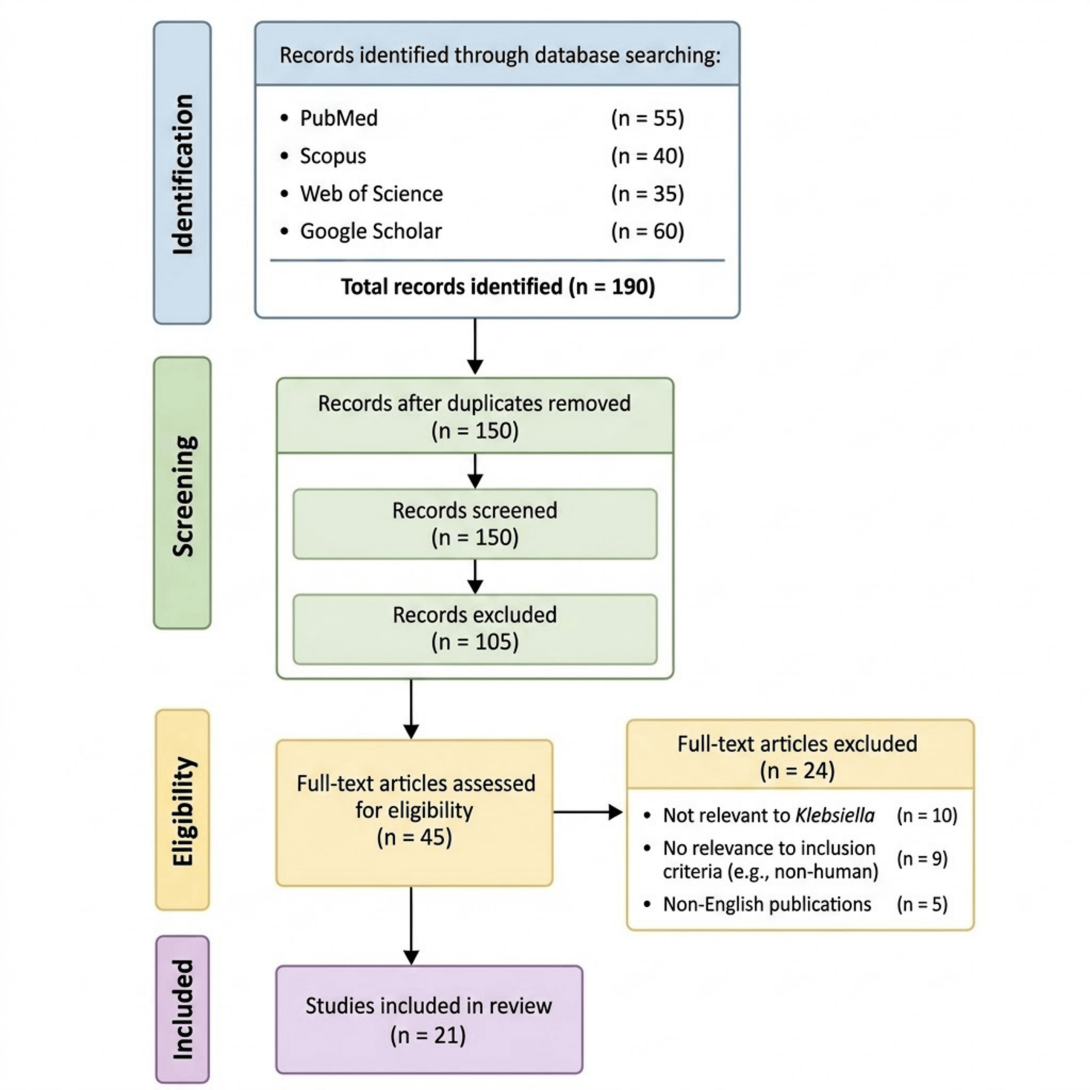
Article selection and screening process n=number of articles

A structured narrative literature review was conducted to identify and synthesize relevant evidence on *Klebsiella* species, focusing on epidemiology, virulence factors, antimicrobial resistance, laboratory diagnosis, and treatment. The review process was designed in alignment with the PRISMA principles to enhance transparency and reproducibility. A comprehensive search was performed across electronic databases, including PubMed, Google Scholar, and Scopus, using combinations of keywords, such as “*Klebsiella pneumoniae,*” “*Klebsiella* species,” “multidrug resistance,” “extended-spectrum beta-lactamase (ESBL),” “AmpC,” “carbapenem resistance,” “virulence factors,” and “nosocomial infections.” Studies published between 2006 and 2026 were considered to ensure the inclusion of both foundational and recent evidence. Articles were selected based on relevance to the topic. Inclusion criteria comprised human-related studies focusing on *Klebsiella* species, published in English, and addressing virulence, antimicrobial resistance, diagnosis, or treatment, including both original research and review articles. Exclusion criteria included studies not primarily focused on *Klebsiella* species, non-human studies, non-English publications, and duplicate or irrelevant articles. A total of 21 studies were included following the screening of titles, abstracts, and full texts. The study selection process is presented using a PRISMA-style flow diagram (Figure 1). Risk of bias was assessed qualitatively, and the included studies were considered to have an overall low to moderate risk of bias due to variability in study design, sample size, and geographic distribution. The selected literature was critically analyzed and synthesized to provide a comprehensive overview of the clinical and microbiological aspects of *Klebsiella* infections.

## Review

*Klebsiella *spp. are widely distributed in environmental sources and commonly colonize the mucosal surfaces of healthy mammals. *K. pneumoniae* and *K. oxytoca* are important opportunistic pathogens associated with community- and hospital-acquired infections, particularly in immunocompromised patients [1].

*Klebsiella* species can cause both community-acquired and nosocomial infections, including respiratory tract, urinary tract, and wound infections [2,3].

Many human-causing species, including *Klebsiella pneumoniae, Klebsiella ozaenae, Klebsiella rhinoscleromatis, Klebsiella oxytoca, *and *Klebsiella aerogenes*, are members of the genus *Klebsiella*. The two main pathotypes of *Klebsiella *species, the hv and MDR clones, account for a significant portion of infections [2].

Because the two branches' strains have distinct genetic backgrounds, these were regarded as non-overlapping. However, it has been demonstrated that *Klebsiella *species can acquire genetic components and mutations that give antibiotic resistance and/or virulence features.

This eventually results in the creation of convergent clones known as MDR and MDRhv *Klebsiella *spp. MDR-hv dual hypervirulence. Resistance to antibiotics is exhibited by *Klebsiella *species, which are thought to continue developing to produce strains with different phenotypes. Numerous MDR-hv *Klebsiella *species strains that have developed via various methods have been recorded in numerous publications from different continents worldwide. Because of the increase in serious infections and the rising absence of effective therapies, *Klebsiella *bacteria that are MDR-hv have evolved into true superbugs that present grave dangers to the general public's health [2].

History of *Klebsiella*

*Klebsiella *species were first described in 1882 by the German pathologist Carl Friedländer, who isolated a capsulated bacillus from the lungs of patients who had died of severe pneumonia [4]. Because of this discovery, the organism was initially known as Friedländer’s bacillus and was believed to be a major cause of lobar pneumonia [4]. As bacterial classification systems developed, the organism was reclassified into the genus *Klebsiella*, named in honor of Edwin Klebs, a German microbiologist [4].

The genus's initial categorization was mostly based on the diseases produced by the organisms [4]. Accordingly, several species were recognized, including *Klebsiella pneumoniae,* associated with pneumonia; *Klebsiella rhinoscleromatis*, associated with rhinoscleroma; and *Klebsiella ozaenae*, associated with ozena or atrophic rhinitis [4]. With the advancement of biochemical testing and taxonomic studies, these organisms were later grouped more closely within the genus, and some were reclassified as subspecies or variants of *K. pneumoniae* [4].

*Klebsiella *species were subsequently placed in the family Enterobacteriaceae, as they share morphological and biochemical characteristics with other enteric gram-negative bacilli [4]. Over time, they came to be recognized not only as pathogens causing specific diseases but also as opportunistic organisms that form a component of the digestive tract's typical flora and respiratory mucosa [4]. Koneman highlights the growing role of these organisms as important nosocomial pathogens, connected to UTIs, pneumonia, septicemia, and wound infections, especially among hospitalized and immunocompromised individuals [4].

Taxonomical classification of *Klebsiella *species 

Gram-negative, encapsulated, nonmotile, rod-shaped, and oxidase-negative bacteria belong to the *Klebsiella *genus [5]. Trevisan (1885) named strains of this genus in honor of German scientist Edwin Klebs (1834-1913) when they were first found in the late nineteenth century [5]. The Enterobacteriaceae family, which includes *Escherichia coli,* is the model organism and the infamous human pathogens *Salmonella, Yersinia, Serratia, Enterobacter, Citrobacter, Kluyvera, Leclercia, Raoultella, Cronobacter,* etc., includes *Klebsiella *[5]. With an isolation rate of about 85%, *K. pneumoniae*, the type species of the *Klebsiella *genus, is very common in clinical samples [5].

*Klebsiella *species 

Among the most resilient opportunistic infections, *Klebsiella *species* *are the well-researched microorganisms in the medicine field and in the industry [6]. Many strains of the genus *Klebsiella *have developed into a significant global concern to clinical and public health [5]. The emergence of MDR-hv *Klebsiella *spp. has been associated with an increase in severe infections and limited treatment options [5]. In addition to being common in natural settings, *Klebsiella oxytoca *and *Klebsiella pneumoniae *are commensals in the human gut [6]. *K. variicola* was first discovered to be a pathogen in people and an endophyte in plants [7]. *Klebsiella aerogenes* has been linked to blood infections, gastrointestinal, urinary, respiratory, and lumbar spine infections, according to clinical research [8].

Pathogenicity and virulence factors of Klebsiella spp. 

The major virulence factors of *Klebsiella *are summarized in Table 1.

**Table 1:** Virulence factors of Klebsiella spp. Virulence factors of *Klebsiella pneumoniae* and their roles in pathogenesis. LPS: lipopolysaccharide; UTI: urinary tract infection

Virulence factor	Description	Mode of action	Pathogenic importance
Capsular polysaccharide (CPS)	Thick outer polysaccharide layer with multiple K antigen types, especially K1 and K2	Protects bacteria from phagocytosis and complement-mediated killing by preventing C3b binding	Primary determinant of virulence; K1 and K2 strains commonly cause severe infections such as pneumonia, septicemia, and UTIs
Capsular sugar composition	Presence or absence of mannose or rhamnose residues in the capsule	Mannose-containing capsules are recognized by macrophage lectin receptors, leading to non-opsonic phagocytosis	Strains lacking these sugars escape macrophage recognition and show enhanced virulence
Type 1 fimbriae	Mannose-sensitive surface appendages	Mediate attachment to mannose-rich glycoproteins on epithelial cells	Facilitates colonization of the urinary and respiratory mucosa; expression is downregulated during tissue invasion
Type 3 fimbriae	Mannose-resistant adhesive structures	Enable adherence to damaged tissues, basement membranes, and indwelling medical devices	Important for persistence in catheter-associated urinary tract infections
Additional adhesins	Non-fimbrial and fimbrial adhesins (e.g., CF29K, KPF-28)	Promote adherence to intestinal epithelial cells	Likely involved in gastrointestinal colonization; some are associated with antibiotic-resistant strains
Lipopolysaccharide (O antigen)	Outer membrane component with limited O serotypes	Interferes with complement-mediated bacterial lysis by inhibiting the development of membrane attack complexes	Increases the risk of serum resistance and systemic infections
Serum resistance mechanisms	Capsule, smooth LPS, and outer membrane proteins	Reduce complement activation and deposition on the bacterial surface	Allows survival in the bloodstream and promotes invasive disease
Environment-dependent LPS variation	Structural changes in LPS influenced by osmolarity	High osmolarity enhances smooth LPS formation and serum resistance	Explains variation in virulence at different body sites
Enterobactin	Phenolate-type iron-chelating compound	Sequesters iron from host iron-binding proteins	Widely produced; role in virulence remains unclear
Aerobactin	Plasmid-encoded hydroxamate siderophore	Efficiently acquires iron from host cells and can be reused	Strongly associated with increased virulence, particularly in K1 and K2 strains
Aerobactin receptors	Surface receptors for aerobactin	Permit the utilization of aerobactin produced by other bacteria	Provides a competitive advantage during mixed infections
Cytotoxins	Occasionally produced toxins	Cause limited host cell damage	Minor contribution to disease
Enterotoxins	Rarely expressed	Induce intestinal fluid secretion	Limited pathogenic role
Hemolysin	Infrequently produced toxin	Causes red blood cell lysis	Minimal importance in virulence
Endotoxin activity (lipid A)	Toxic component of LPS	Triggers strong inflammatory and immune responses	Responsible for endotoxemia and septic shock
Biofilm formation	Mediated mainly by type 3 fimbriae	Enhances bacterial persistence on surfaces	Plays a key role in device-associated infections

Clinical significance of *Klebsiella *spp.

Clinical Significance of Klebsiella pneumoniae 

Recently, the World Health Organization discovered *K. pneumoniae *as among the most critical opportunistic pathogens for which the development of new antibiotics is urgently needed [6]. These strains, which are a part of the gastrointestinal flora, have the potential to cause serious infections while also exhibiting increased resistance to first-line antibiotics, such as cephalosporins, fluoroquinolones, and aminoglycosides, as well as recently documented resistance to tigecycline and colistin. Furthermore, it is often known that *Klebsiella *species play a significant role in the transmission of carbapenem resistance genes to other species [6]. Although treating nosocomial infections with carbapenem-resistant *K. pneumoniae* (CRKP) is difficult, the strain's pathogenicity is being further enhanced by additional β-lactamase-encoding genes, making the issue more severe and increasing resistance to penicillins, aztreonam, and first-, second-, and third-generation cephalosporins (apart from clavulanic acid) [6]. The fimbrial adhesions (mannose-sensitive type 1 and type 3), non-fimbrial adhesion (CF29K factor), siderophores, and surface saccharides (capsule, which describes capsular serotypes; there are currently 79 recognized serotypes, and LPS, which describes O-antigens) are the main components of *K. pneumoniae *virulence [6]. Other virulence factors, such as iron transport systems, genes linked to allantoin metabolism, or porins, have recently been discovered. However, at present, there are not enough studies to define them appropriately [6]. These facultative anaerobes are between 0.3 and 1.8 µm in size, readily ferment lactose, and form mucoid colonies because of their capsules, which are crucial to *Klebsiella *strains' defense mechanisms as they provide resistance to phagocytosis or lysis [6].

Clinical Significance of Klebsiella oxytoca 

In terms of taxonomy, *Klebsiella oxytoca* (Lautrop, 1956) was first identified and described by Flugge in 1886 as a strain of *Bacillus oxytocus perniciosus *isolated from sour milk. It was also mentioned as a microorganism linked to Musca domestica, as *Bacterium oxytocum*, a clinical isolate from an abdominal infection (actinomycosis), and as *Aerobacter oxytocum *in honor of Trevisan or Bergey (1923) [6]. Using DNA-DNA hybridization, Jain et al. identified the* Oxytocum* group as distinct from the *Klebsiella *genus in 1974. Later, in 1980, *K. oxytoca* attracted special attention after certain strains were discovered to have a beta-lactam resistance characteristic (Granier et al.) [6]. *K. oxytoca *is an opportunistic pathogen that causes pneumonia, septicemia, and UTIs in humans. It has also emerged as a significant bacterium in hospital-acquired infections, but it can also be a beneficial microorganism, an endophytic nitrogen-fixing bacterium, and a microorganism that produces exopolysaccharides [6]. Despite being widely distributed in the environment, it is common to isolate strains from human or animal skin, mucosal membranes, oropharynx, and intestine, in addition to from clinical patients and medical facilities [6]. *K. oxytoca* is a multi-resistant species to antibiotics with a wide spectrum of activity, such as cephalosporins (ceftriaxone, cefotaxime, or ceftazidime) and aztreonam (monobactam). Additionally, its resistance to non-beta-lactam antibiotics was found among clinical isolates, and it is believed to be higher than that of *K. pneumoniae. *Additionally, it resists the commonly used antiseptic chlorhexidine [6]. Consequently, this microbe produces a K1 enzyme (KOXY type), a molecular class, and is inherently resistant to amino- and carboxypenicillins. The presence of a molecular class-A serine beta-lactamase, KPC, which is typically found in *K. pneumoniae*, is still susceptible to clavulanic acid and tazobactam, and its resistance to carbapenems (due to the production of carbapenemases, i.e., imipenemases), such as imipenem, ertapenem, and meropenem (while remaining susceptible to tigecycline, ciprofloxacin, and colistin) [6].

Clinical Significance of Klebsiella variicola 

First described in 2004, *Klebsiella variicola* was later identified as *Klebsiella quasipneumoniae* in 2014 (with two subspecies: *K. quasipneumoniae subsp. quasipneumoniae *and *K. quasipneumoniae subsp. similipneumoniae) *and *Klebsiella quasivariicola *(which is still pending validation) in 2017 [7]. 2019 saw the description of *Klebsiella africanensis*, a new species of bacteria, and *Klebsiella variicola subsp. tropicalensis, *a *subspecies *of *K. variicola *[7]. The taxonomy of the genus *Klebsiella*, which is known as the *Klebsiella pneumoniae* complex, has been broadened by the description of several additional bacterial species [7]. Since its description, *K. variicola *has been the subject of numerous international publications discussing its significance; in fact, it is regarded as an emergent human pathogen [7]. *K. variicola* is a gram-negative, facultative anaerobic, nonspore-forming, nonmotile rod-shaped bacterium that forms smooth, convex, and round colonies, just like other *Klebsiella *species [7]. *K. variicola* was first found to be a pathogen in people and an endophyte in plants [7]. Furthermore, *K. variicola *is regarded as a pathogen in plants and animals and a symbiont in insects. Additionally, *K. variicola* has been discovered in several environmental sources [7]. *K. variicola* is a pathogen that affects humans and has been isolated from a variety of clinical materials, such as blood, tracheal aspirates, various secretions, respiratory tract infections and UTIs, and surgical wounds [7]. *K. variicola*'s estimated prevalence varies greatly; it was first reported to be 8%, but over time, it has fluctuated between 1.8% and 24.4% in clinical settings [7]. Also, 24.4% is the greatest percentage that has been documented thus far, and it came from bloodstream infections [7].

Clinical Significance of Klebsiella aerogenes 

*Klebsiella aerogenes*, formerly known as *Enterobacter aerogenes*, belongs to the Enterobacteriaceae family. It is present in the human digestive system and is extensively dispersed throughout the environment. *K. aerogenes* belongs to the Enterobacteriaceae family and is a facultative anaerobic gram-negative bacterium [8]. It is widely dispersed among the surroundings and is found in the human gastrointestinal tract, also being a common opportunistic pathogen in hospitals [8]. When the host immune system is compromised or the intestinal mucosa is damaged, it may cause infection of the respiratory, circulatory, or urogenital system [8]. In recent years, despite increasing reports on the pathogenicity and drug resistance of *Klebsiella pneumoniae *and *Escherichia coli*, there have only been a few reports on *K. aerogenes *[8]. Previous clinical reports on this bacterium were mainly of urinary tract, respiratory system, blood infections, and digestive system; it can also infect the lumbar spine [8]. In contrast to other species of Enterobacteriaceae, *K. aerogenes* is more likely to cause septic shock or even death in patients [8].

Diseases and clinical manifestations caused by *Klebsiella *species

Being among the most resilient opportunistic infections, *Klebsiella *species are among the well-researched microorganisms in the medical profession [6]. *Klebsiella *species are significant opportunistic pathogens linked to numerous clinical illnesses [4]. These organisms commonly cause infections of the respiratory tract, including severe lobar pneumonia, bronchopneumonia, lung abscess, and ventilator-associated pneumonia, particularly in hospitalized or debilitated patients [4]. They are also frequent causes of urinary tract infections such as cystitis, pyelonephritis, and catheter-associated UTIs [4]. In addition, *Klebsiella *species are responsible for bloodstream infections, leading to bacteremia and septicemia, especially among immunocompromised individuals [4]. Wound and soft-tissue infections, including surgical site infections and cellulitis, are also reported. Intra-abdominal infections, such as liver abscess, peritonitis, and biliary tract infections, may occur, and central nervous system involvement can lead to meningitis, particularly in neonates or following neurosurgical procedures [4]. Certain species are associated with specific clinical syndromes, including rhinoscleroma caused by *Klebsiella rhinoscleromatis* and ozena (atrophic rhinitis) caused by *Klebsiella ozaenae* [4].

Multidrug resistance in *Klebsiella *species 

For epidemiologic purposes, multidrug-resistant organisms (MDROs) are defined as microorganisms, predominantly bacteria, that are resistant to one or more classes of antimicrobial agents [9]. The evolutionary pressure of survival drives the emergence of drug resistance and thereby poses a major challenge to modern medicine [10]. Resistance threatens the longevity of drugs and restricts treatment options for patients, with high prevalence [10]. Antibiotic resistance has been increasing at alarming rates, with hardly any novel antibiotics in clinical development [9]. Once a mainstay of antibiotics because of their capacity to disrupt the bacterial cell wall, β-lactams have been severely compromised by resistance [10]. *Klebsiella *species have been linked to different infection types, and a particularly concerning trend is the rise of MDR strains, especially those associated with hospital-acquired infections [11]. Strains of bacteria that produce *Klebsiella *carbapenemases and extended-spectrum β-lactamases (ESBL) are emerging rapidly as significant contributors to MDR infections globally [11]. Bacterial isolates that carry these enzymes can break down a wide range of β-lactams, such as penicillins, cephalosporins, carbapenems, and monobactams [11]. Numerous cases have shown resistance to nearly all antibiotics [11]. Strains of *Klebsiella *cause a broad range of diseases in humans [11]. These bacteria have emerged as significant pathogens in hospital-acquired infections [11]. Epidemic and endemic hospital-acquired infections attributed to *Klebsiella *species are major contributors to morbidity and mortality [11]. *K. pneumoniae *and *K. oxytoca* are the two most prevalent species of *Klebsiella *that cause infections in humans [11]. *K. variicola *was initially identified as an endophyte in plants as well as a pathogen in humans [7]. Clinical reports on* K. aerogenes *were mainly of urinary tract, digestive system, respiratory system, and blood infections; it can also infect the lumbar spine [8].

Mechanisms of antibiotic resistance in *Klebsiella *species 

Although many mechanisms are conserved across Enterobacterales, this section primarily focuses on *Klebsiella *spp., and other bacteria are only brought up when they provide canonical mechanistic examples relevant to *Klebsiella *[12]. Antibiotic resistance in *Klebsiella *spp. is best understood as a layered phenotype, in which multiple mechanisms combine to reduce adequate drug exposure and/or neutralize the action of drugs [12]. Intrinsic resistance, acquired resistance, and adaptive resistance are the types of antibiotic resistance in *Klebsiella *species [12]. Different resistance mechanisms of *Klebsiella *are outlined in Table 2.

**Table 2:** Mechanisms of antibiotic resistance in Klebsiella spp. Types of antibiotic resistance in *Klebsiella pneumoniae* and their underlying mechanisms. ESBL: extended-spectrum β-lactamase; KPC: *Klebsiella pneumoniae* carbapenemase; NDM: New Delhi metallo-β-lactamase; OXA: oxacillinase

Type of resistance	Definition	Main mechanisms	Key examples in *Klebsiella*
Intrinsic resistance	Natural, chromosomally encoded resistance is present in all members of the species	Outer membrane permeability barrier, porin regulation, efflux pumps, chromosomal β-lactamase	SHV-1-mediated ampicillin resistance; porins OmpK35 and OmpK36; efflux pumps AcrAB-TolC and OqxAB; LPS barrier
Acquired resistance	Resistance gained through mutations or horizontal gene transfer	Plasmids, transposons, integrons carrying resistance genes; enzyme production; porin loss or modification	ESBLs (CTX-M, TEM, SHV variants); carbapenemases (KPC, NDM, OXA-48); plasmid-mediated resistance genes; porin loss with β-lactamases
Adaptive resistance	Temporary resistance developed in response to environmental stress	Upregulation of efflux pumps, altered porin expression, stress response systems	Increased expression of AcrAB-TolC or OqxAB; reduced porin expression during antibiotic exposure

β-Lactamase-Mediated Resistance in Klebsiella 

MDR enterobacteriaceae is emerging globally as among the most serious health problems, leading to treatment failure of both community - acquired as well as nosocomial infections. *Klebsiella *is classified under the Enterobacteriaceae family [5]. The acquired resistance to various beta-lactams in *Klebsiella *is a growing problem worldwide. This resistance is primarily due to the development of diverse beta-lactamases. Carbapenems are the most favored antibiotics commonly utilized to cure infections caused by MDR Enterobacteriaceae. The improper and needless use of β-lactam medications, which results in the selection of various mutant forms of β-lactamases, is one of the main sources of bacterial resistance. The most concerning resistance mechanisms at the moment are ESBLs, AmpC, and metallo-β-lactamase (MBL), which have an uncontrollable effect on antimicrobial therapy. Extended-spectrum β-lactamases (ESBLs) are enzymes that offer defense against cephalosporins, penicillins, and aztreonam by hydrolyzing these antibiotics; however, they are inhibited by β-lactamase inhibitors such as clavulanic acid. ESBL-producing organisms are often MDR and may also show resistance to aminoglycosides, cotrimoxazole, tetracyclines, and fluoroquinolones. AmpC β-lactamases hydrolyze cephalosporins, cephamycins, aminopenicillins, and monobactams, and are less inhibited by clavulanic acid. Carbapenems were once considered the drugs of choice for infections caused by ESBL- and AmpC-producing organisms; however, the emergence of MBL producers has led to carbapenem resistance. Carbapenem-resistant Enterobacteriaceae frequently co-produce ESBL, AmpC, or both, often through plasmid-mediated transfer. The co-occurrence of multiple β-lactamases within a single organism can lead to diagnostic as well as therapeutic failures, making it essential to identify these enzymes using specific phenotypic tests rather than relying solely on routine antibiotic susceptibility testing [4,13,14].

Laboratory diagnosis of *Klebsiella *species 

Laboratory diagnosis of *Klebsiella *species involves proper specimen collection, culture, identification, biochemical characterization, and testing for antimicrobial susceptibility [4,15,16]. Clinical specimens depend on the site of infection and commonly include urine, sputum, blood, pus, wound swabs, and other body fluids [4,15,16]. Specimens are inoculated onto routine media such as blood agar and MacConkey agar [4,15,16]. On MacConkey agar, *Klebsiella* species produce large, mucoid, pink lactose-fermenting colonies due to their prominent polysaccharide capsule [4,15,16]. Colonies on blood agar are usually large, grayish, smooth, and mucoid [15-20].

Preliminary identification is based on Gram staining, which shows gram-negative, short, plump bacilli either alone or in pairs [4,15,16]. *Klebsiella *species are non-motile, oxidase-negative, and facultative anaerobes [4,15,16]. Further identification is achieved through biochemical tests [4,15,16]. They are typically indole variable (indole positive in *K. oxytoca *and negative in *K. pneumoniae*), urease positive, citrate positive, and Voges-Proskauer positive, while methyl red is negative [4,15,16]. They ferment glucose and lactose with the generation of acid and are non-motile, which distinguishes them from other Enterobacteriaceae [4,15,16].

In modern laboratories, automated identification systems, such as VITEK, MicroScan, and MALDI-TOF mass spectrometry, are frequently employed for rapid and accurate species identification [4,15,16]. Antimicrobial susceptibility testing is performed using common techniques, including the Kirby-Bauer disc diffusion or automated systems, following standard guidelines [4,15,16]. Special phenotypic tests may also be performed to detect ESBLs, AmpC, or synthesis of carbapenemase, which is essential for guiding appropriate therapy [4,15,16]. These combined methods form the basis of routine laboratory diagnosis of *Klebsiella *infections [4,15,16,17]. Culture and biochemical characteristics of *Klebsiella *are shown in Table 3.

**Table 3:** Culture and biochemical characteristics of Klebsiella spp. Comparative microbiological and biochemical characteristics of clinically significant *Klebsiella *species. MR: methyl red; VP: Voges-Proskauer; H₂S: hydrogen sulfide

Characteristic	K. pneumoniae	K. oxytoca	K. variicola	*K. aerogenes *(formerly Enterobacter aerogenes)	K. ozaenae	K. rhinoscleromatis
Gram stain	Gram-negative bacilli	Gram- negative bacilli	Gram- negative bacilli	Gram-negative bacilli	Gram-negative bacilli	Gram-negative bacilli
Motility	Non-motile	Non-motile	Non-motile	Motile	Non-motile	Non-motile
Capsule	Prominent	Prominent	Present	Less prominent	Present	Prominent
MacConkey agar	Large, mucoid, lactose-fermenting (pink) colonies	Mucoid, lactose-fermenting (pink) colonies	Mucoid, lactose-fermenting (pink) colonies	Lactose-fermenting (pink) colonies	Small, less mucoid lactose- fermenting (pink) colonies	Mucoid lactose-fermenting (pink) colonies
Blood agar	Large, gray, mucoid colonies	Large, gray, mucoid colonies	Mucoid colonies	Smooth colonies	Smaller colonies	Mucoid colonies
Oxidase	Negative	Negative	Negative	Negative	Negative	Negative
Catalase	Positive	Positive	Positive	Positive	Positive	Positive
Indole	Negative	Negative	Usually negative	Negative	Negative	Negative
Methyl red (MR)	Negative	Negative	Negative	Variable/negative	Negative	Negative
Voges-Proskauer (VP)	Positive	Positive	Positive	Positive	Positive	Positive
Citrate utilization	Positive	Positive	Positive	Positive	Variable	Variable
Urease	Positive	Positive	Positive	Weak/variable	Positive	Positive
H_2_S production	Negative	Negative	Negative	Negative	Negative	Negative
Lysine decarboxylase	Positive	Positive	Positive	Negative	Negative	Negative
Glucose fermentation	Acid + gas	Acid + gas	Acid + gas	Acid + gas	Acid + gas	Acid + gas
Lactose fermentation	Positive	Positive	Positive	Positive	Positive (slow)	Positive

Treatment

The patterns of antibiotic resistance, corresponding Clinical and Laboratory Standards Institute (CLSI)-based antimicrobial susceptibility testing (AST) interpretations, and commonly used treatment options for *Klebsiella* species are summarized in Table 4. ESBL-producing isolates exhibit resistance to third-generation cephalosporins but generally remain susceptible to carbapenems, which are considered the treatment of choice, particularly in severe infections. Although some ESBL-producing isolates may appear susceptible to piperacillin-tazobactam based on CLSI breakpoints, its use remains controversial due to the potential risk of in-treatment failure, especially in serious infections. AmpC-producing isolates demonstrate resistance to several β-lactams, including cephamycins, thereby limiting therapeutic options. Carbapenem-resistant *Klebsiella* (CRE) infections require treatment with newer or last-resort agents. Pan-resistant isolates pose significant therapeutic challenges and often necessitate individualized combination therapy approaches [18].

**Table 4:** Treatment. Antibiotic resistance patterns in *Klebsiella *spp. with corresponding CLSI AST interpretation and treatment options. CLSI: Clinical and Laboratory Standards Institute; AST: antimicrobial susceptibility testing; ESBL: extended-spectrum β-lactamase

Resistance pattern in *Klebsiella*	CLSI laboratory findings (AST result)	CLSI reporting guidance	Common antibiotics used in treatment
Susceptible isolates	Susceptible to third-generation cephalosporins, fluoroquinolones, aminoglycosides, etc.	Report drugs as S according to CLSI breakpoints	Ceftriaxone, cefotaxime, cefepime, piperacillin-tazobactam, amoxicillin-clavulanate, ciprofloxacin, levofloxacin, gentamicin, amikacin, trimethoprim-sulfamethoxazole
ESBL-producing *Klebsiella*	Resistant to cefotaxime, ceftazidime, and ceftriaxone; usually susceptible to carbapenems	Report results according to breakpoints; ESBL confirmation optional	Carbapenems (first-line): imipenem, meropenem, doripenem, ertapenem; if susceptible and in non-severe infections: piperacillin-tazobactam (use with caution), amikacin, tigecycline, trimethoprim-sulfamethoxazole
AmpC-producing *Klebsiella*	Resistance to cephamycins (cefoxitin) and some cephalosporins	Report based on AST results	Carbapenems (imipenem, meropenem), cefepime (if susceptible), amikacin, fluoroquinolones (if susceptible), trimethoprim-sulfamethoxazole (if susceptible)
Carbapenem-resistant *Klebsiella* (CRE)	Resistant to imipenem, meropenem, or ertapenem	Report as resistant; perform carbapenemase testing if required	Colistin (polymyxin E), polymyxin B, tigecycline, aminoglycosides (amikacin), fosfomycin, newer agents: ceftazidime-avibactam, meropenem-vaborbactam
Pan-resistant isolates	Resistant to all tested antibiotics	Report all as resistant	Combination therapy based on susceptibility: colistin, tigecycline, fosfomycin, aminoglycosides (if any activity remains)

## Conclusions

Because of its many virulence characteristics and quickly developing antibiotic resistance mechanisms, *Klebsiella pneumoniae* continues to be a significant global disease. In host colonization, immune evasion, and disease progression, structural elements such as the capsular polysaccharide, lipopolysaccharide, fimbriae, and iron acquisition mechanisms are crucial. ESBL-producing and carbapenem-resistant isolates are among the increasingly common MDR infections that have severely limited treatment options and complicated clinical care. Accurate diagnosis and successful therapy selection depend on a thorough understanding of these pathogenic processes and resistance profiles.

To counter the increasing threat of *Klebsiella *infections, it is imperative that ongoing surveillance be conducted, new antimicrobial drugs be developed, and effective infection control measures be put in place. Early detection and patient outcomes may be enhanced by developments in targeted medicines and molecular diagnostics. Global antimicrobial stewardship initiatives and prudent antibiotic use are also essential for reducing the onset and spread of resistance. To lower the clinical burden and enhance the treatment of infections linked to *Klebsiella*, these strategies must be strengthened.
